# Role of 3D quantitative tumor analysis for predicting overall survival after conventional chemoembolization of intrahepatic cholangiocarcinoma

**DOI:** 10.1038/s41598-021-88426-x

**Published:** 2021-04-29

**Authors:** Irvin Rexha, Fabian Laage-Gaupp, Julius Chapiro, Milena Anna Miszczuk, Johanna Maria Mijntje van Breugel, MingDe Lin, Menelaos Konstantinidis, Rafael Duran, Bernhard Gebauer, Christos Georgiades, Kelvin Hong, Nariman Nezami

**Affiliations:** 1 Department of Radiology and Biomedical Imaging, Yale University School of Medicine, New Haven, CT USA; 2 Department of Diagnostic and Interventional Radiology, Charité Universitätsmedizin, Campus Virchow Klinikum, Berlin, Germany; 3 Division of Biostatistics, Dalla Lana School of Public Health, University of Toronto, Toronto, ON Canada; 4 Division of Vascular and Interventional Radiology, Russel H. Morgan Department of Radiology and Radiological Sciences, Johns Hopkins University School of Medicine, Baltimore, MD USA; 5 Division of Interventional Radiology and Image-Guided Medicine, Department of Radiology and Imaging Sciences, Emory University School of Medicine, 1364 Clifton Rd, Suite AG05, Atlanta, GA 30322 USA

**Keywords:** Cancer imaging, Liver cancer

## Abstract

This study was designed to assess 3D vs. 1D and 2D quantitative tumor analysis for prediction of overall survival (OS) in patients with Intrahepatic Cholangiocarcinoma (ICC) who underwent conventional transarterial chemoembolization (cTACE). 73 ICC patients who underwent cTACE were included in this retrospective analysis between Oct 2001 and Feb 2015. The overall and enhancing tumor diameters and the maximum cross-sectional and enhancing tumor areas were measured on baseline images. 3D quantitative tumor analysis was used to assess total tumor volume (TTV), enhancing tumor volume (ETV), and enhancing tumor burden (ETB) (ratio between ETV and liver volume). Patients were divided into low (LTB) and high tumor burden (HTB) groups. There was a significant separation between survival curves of the LTB and HTB groups using enhancing tumor diameter (*p* = 0.003), enhancing tumor area (*p* = 0.03), TTV (*p* = 0.03), and ETV (*p* = 0.01). Multivariate analysis showed a hazard ratio of 0.46 (95%CI: 0.27–0.78, *p* = 0.004) for enhancing tumor diameter, 0.56 (95% CI 0.33–0.96, *p* = 0.04) for enhancing tumor area, 0.58 (95%CI: 0.34–0.98, *p* = 0.04) for TTV, and 0.52 (95%CI: 0.30–0.91, *p* = 0.02) for ETV. TTV and ETV, as well as the largest enhancing tumor diameter and maximum enhancing tumor area, reliably predict the OS of patients with ICC after cTACE and could identify ICC patients who are most likely to benefit from cTACE.

## Introduction

Intrahepatic cholangiocarcinoma (ICC) is a neoplasm of the biliary tract’s epithelial cells^1–5^. It accounts for 10–20% of all cholangiocarcinomas and is the second most common primary liver cancer^1,4–7^. Patients with ICC are commonly diagnosed at advanced stages of the disease due to unspecific symptoms^4,5,7,8^. Therefore, the survival rates are dismal, with an estimated median overall survival (MOS) of 3 to 8 months and fewer than 10% of patients surviving five years after diagnosis^8,9^. Surgery still remains the only curative treatment option; however, only 25–35% of ICC patients are eligible for surgical resection at the time of diagnosis^4,5,9^. Systemic chemotherapy using gemcitabine and cisplatin has limited efficacy, with a MOS of 11–12 months^4,8,10,11^. Within the last two decades, lipiodol-based conventional transarterial chemoembolization (cTACE) has emerged as a palliative treatment option. It is increasingly used in combination with or as second-line treatment after systemic chemotherapy^5,6,8,9,12^. One of the landmark studies in ICC patients demonstrated favorable outcomes for cTACE with a MOS of 12.2 months for the cTACE patients improving to 22 months in responders compared to a MOS of 3.3 months for the best supportive care therapy^13^.

Given the availability of different treatment options for unresectable ICC, it is crucial to precisely assess the tumor burden and evaluate the treatment outcomes for a more personalized treatment plan and potentially improved outcomes^5,14–16^. Although 1-dimensional (1D) and 2-dimensional (2D) tumor assessment methods have been implemented in clinical practice for several decades, these methods have been continuously modified due to subjectivity, lack of reproducibility, and incomplete representation of the tumor^17,18^. Therefore, 3-dimensional (3D) quantitative tumor analysis methods were introduced as a potential solution to address the imprecision in tumor burden assessment^18–20^. The rationale of 3D quantitative tumor analysis on post-contrast MRI is based on the assumption that a viable tumor can be delineated as the enhancing component and used as a quantitative surrogate for the extent of active disease and ultimately as an indicator for tumor response post cTACE. Accordingly, the outcome of cTACE can be visualized and assessed as a decrease in tumor enhancement on MRI, as successful cTACE and locoregional chemotherapy delivery are expected to induce tumor necrosis^21–24^.

Predicting therapeutic outcomes before cTACE and its potential efficacy is important and would allow for a more accurate and individualized patient allocation to this therapy. Thus, this study aimed to evaluate the performance of 3D vs. 1D and 2D methods for quantitative tumor analysis of contrast-enhanced MRI for the prediction of overall survival (OS) in patients with ICC who underwent cTACE.

## Results

### Patient characteristics and clinical outcome

**Figure 1:**
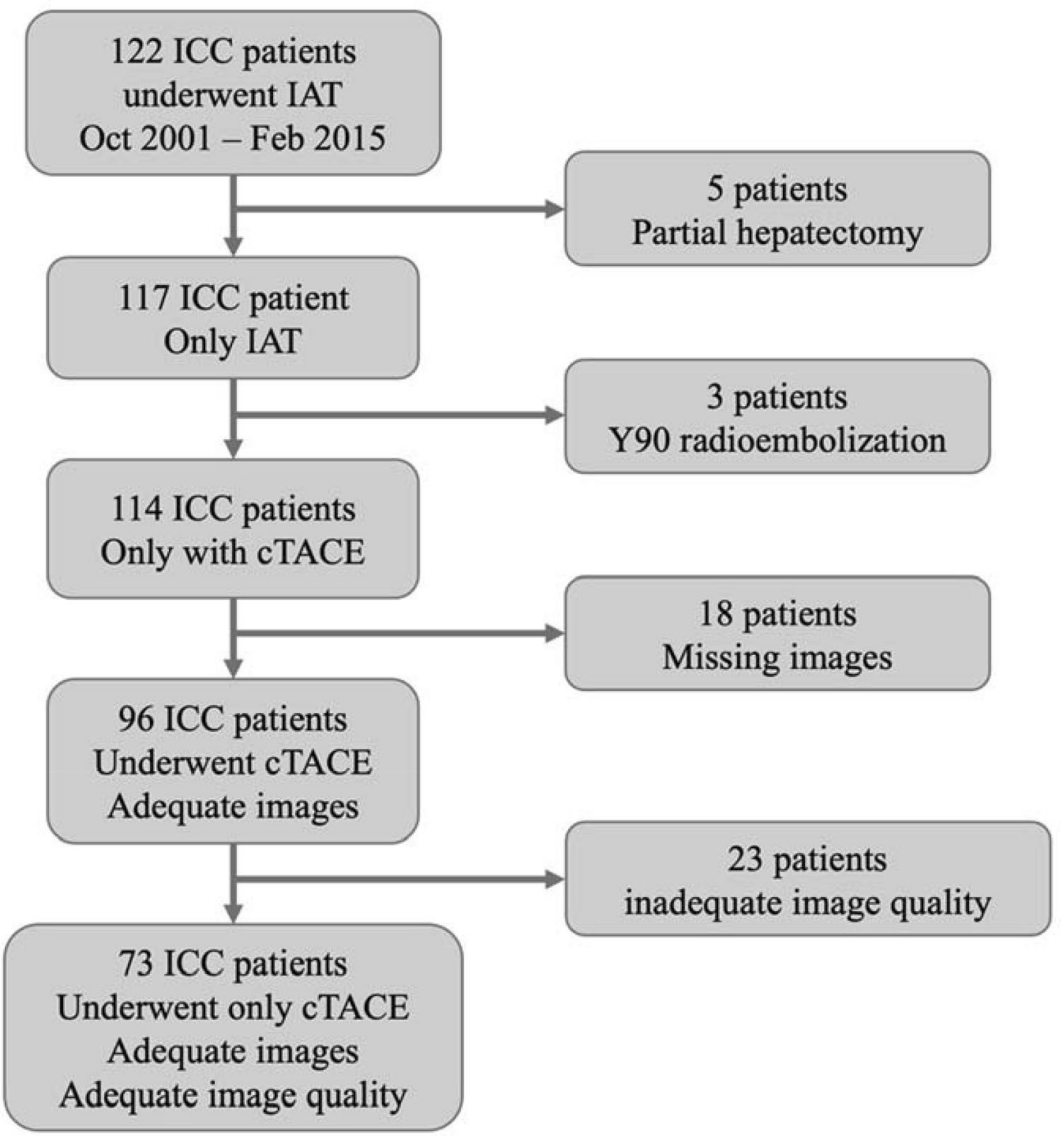
Study flowchart showing number of patients excluded based on exclusion criteria; 73 ICC patients included in the final analysis.

Out of 122 patients, 49 did not meet our inclusion criteria. Eight patients were excluded because 5 had prior partial hepatectomy, and 3 had prior Yttrium-90 radioembolization. Forty-one patients had missing images or inadequate/missing MRI sequences; 9 patients were missing baseline imaging, nine patients were missing critical MRI sequences, and 23 patients had severe motion artifacts. After excluding these 49 patients, a total of 73 patients were enrolled into the final analysis. The study flowchart for inclusion is shown in Fig. 1.

The patients’ baseline characteristics are summarized in Table 1. Mean patient age was 60.32 ± 12.04 years (range 29–83 years), and the MOS was 11 months (range 0.6–52.2 months; 95%CI: 7–14 months). Seven patients had cirrhosis. Primary sclerosing cholangitis was the most common risk factor, affecting 19 (26%) of the included patient cohort. The majority of the ICC patients had Eastern Cooperative Oncology Group (ECOG) scores of 0 or 1 and a Child–Pugh Class of A. All patients received Gemcitabine/Cisplatin-based systemic therapy prior to cTACE (Range 1–11 cycles). Eleven patients additionally received FOLFOX/FOLFIRI-based second-line chemotherapy. The median time interval between pre-interventional imaging and cTACE was 14 days.

**Table 1:** Patients’ demographic characteristics.

Parameter	N	(%)
**Demographics**		
Number of patients	73	(100)
**Age**		
< 65	43	(58.9)
≥ 65	30	(41.10)
**Gender**		
Female	45	(61.64)
Male	28	(38.36)
**Ethnicity**		
African-American	6	(8.22)
Asian/Pacific-Islander	3	(4.11)
Hispanic	2	(2.74)
Other	8	(10.96)
White	54	(73.97)
**Underlying chronic liver disease**		
HBV	4	(5.48)
HCV	4	(5.48)
HIV	4	(5.48)
Alcohol	5	(6.85)
NASH	8	(10.96)
Cirrhosis	7	(9.59)
Primary sclerosing cholangitis	26	(35.62)
**ECOG score**		
0	55	(75.34)
1	13	(17.81)
2	5	(6.85)
**Child–Pugh class**		
A	62	(84.93)
B	10	(13.70)
C	1	(1.370)
**UICC stage**		
0	0	(0)
IA	0	(0)
IB	4	(5.48)
IIA	12	(16.44)
IIB	25	(34.25)
III	0	(0)
IV	32	(43.84)
Average number of cTACE sessions	1.89	(1–5)

HBV: hepatitis B virus, HCV: hepatitis C virus, HIV: human immunodeficiency virus, NASH: nonalcoholic steatohepatitis, ECOG: Eastern Cooperative Oncology Group, UICC: Union for International Cancer Control, cTACE: conventional transarterial chemoembolization.

### Tumor characteristics

Table 2 summarizes the tumor characteristics evaluated in this study.

**Table 2.** 1D, 2D, and 3D tumor burden assessment.

Assessment technique	Mean ± SD	Range
**1D tumor assessment**		
Mean overall tumor diameter (cm)	12.33 ± 6.60	2.44–36.04
Mean enhancing tumor diameter (cm)	9.35 ± 4.64	3.56–28.21
**2D tumor assessment**		
Mean max. cross-sectional area (cm^2^)	64.31 ± 54.73	2.87–353.53
Mean enhancing tumor area (cm^2^)	31.87 ± 25.77	4.12–110.81
**3D tumor assessment**		
Mean total tumor volume (cm^3^)	629.38 ± 748.65	7.78–3566.81
Mean enhancing tumor volume (cm^3^)	289.44 ± 432.79	0.17–2176.77
Mean enhancing tumor burden (%)	11.63 ± 13.90	0.02–72.87

**Figure 2:**
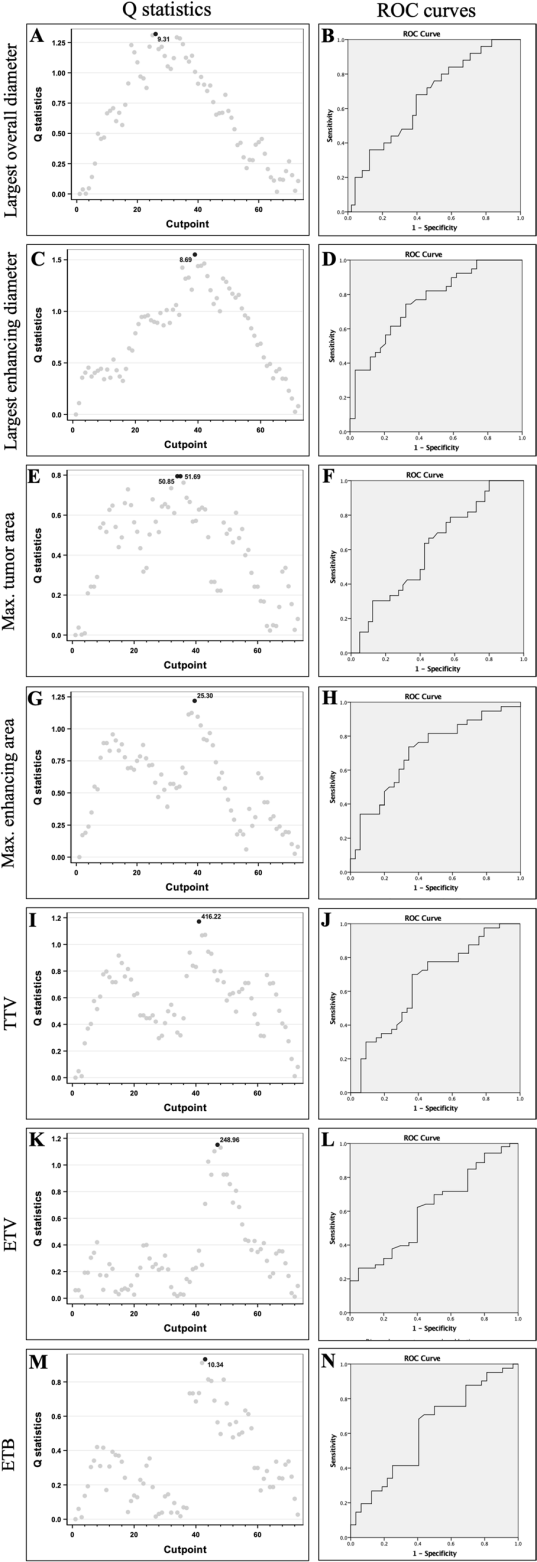
(**A,B**) Based on Q statistics and ROC curve analysis, the cutoff point for the largest overall tumor diameter was determined as 9.3 cm (AUC = 0.664). (**C,D**) Q statistics and ROC curve analysis resulted in a cutoff value of 8.5 cm for the largest enhancing tumor diameter (AUC = 0.759). (**E,F**) Based on Q statistics and ROC curve analysis, the cutoff value for the maximum cross-sectional tumor area was determined as 50.0 cm^2^ (AUC = 0.611). (**G,H**) Q statistics and ROC curve analysis determined a cutoff value of 25.0 cm^2^ for the maximum enhancing tumor area (AUC = 0.708). (**I,J**) ROC curve analysis demonstrated a cutoff value of 410.0 cm^3^ for the total tumor volume (AUC = 0.655). (**K,L**) Based on Q statistics and ROC curve analysis, the cutoff point for the enhancing tumor volume was determined as 275.0 cm^3^ (AUC = 0.612). (**M,N**) Q statistics and ROC curve analysis determined a cutoff value of 10.0% for the enhancing tumor burden (AUC = 0.620).

A total of 419 lesions were measured and analyzed using 1D, 2D, and 3D tumor assessment methods. The mean number of tumor lesions per patient was 1.91. The patients were enrolled into the low tumor burden (LTB) and high tumor burden (HTB) groups according to cutoff values calculated by Q statistic and obtained from receiver operating characteristic (ROC) curve analysis for 1D, 2D, and 3D assessment methods (Table 3; Fig. 2).

**Table 3:** Methods of patient assignment to LTB and HTB in each tumor assessment method.

Assessment method		LTB	HTB	Log-rank *p* value
1D	Overall tumor diameter (cm)	≤ 9.3	> 9.3	0.02
	Enhancing tumor diameter (cm)	≤ 8.5	> 8.5	0.003
2D	Maximum cross-sectional area (cm^2^)	≤ 50	> 50	0.15
	Enhancing tumor area (cm^2^)	≤ 25	> 25	0.03
3D	Total tumor volume (cm^3^)	≤ 410	> 410	0.03
	Enhancing tumor volume (cm^3^)	≤ 275	> 275	0.01
	Enhancing tumor burden (%)	≤ 10	> 10	0.11

LTB: low tumor burden, HTB: high tumor burden, 1D: 1-dimensional, 2D: 2-dimensional, 3D: 3-dimensional.

### Survival analysis

#### 1D tumor assessment

**Figure 3:**
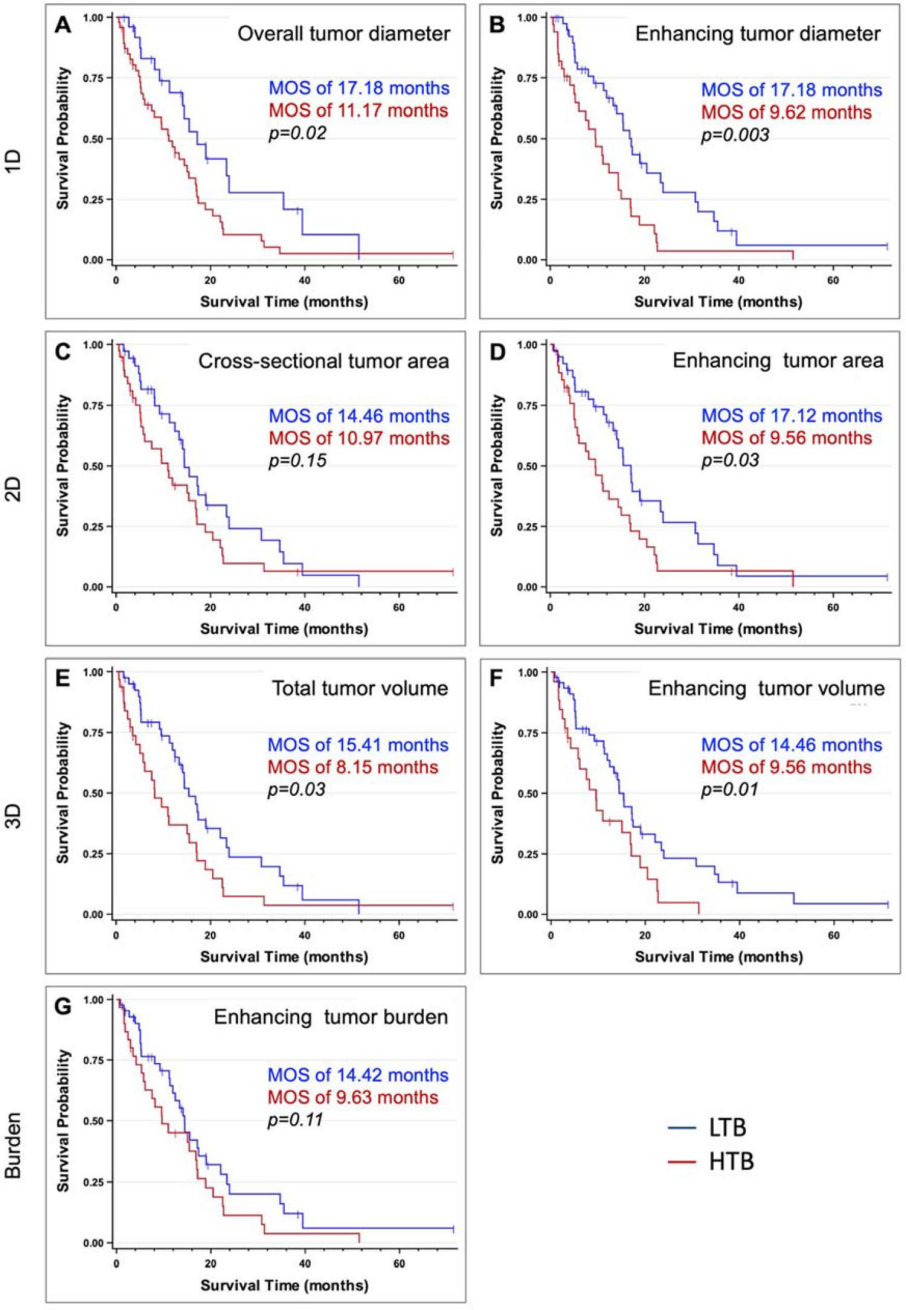
Kaplan–Meier analysis for overall survival calculated for each tumor burden assessment method after categorizing the ICC patients into two groups of low tumor burden (LTB) and high tumor burden (HTB). (**A,B**) Show the 1D-based tumor assessment methods, the largest overall tumor diameter and largest enhancing tumor diameter. (**A**) ICC patients with largest overall tumor diameter of ≤ 9.3 cm were categorized into the LTB group. There was a significant difference between survival curves of the LTB and HTB groups based on the Log-rank test (*p* = 0.02). (**B**) Patients with the largest enhancing tumor diameter of ≤ 8.5 cm were enrolled into the LTB group. A significant separation of survival curves between the LTB and HTB groups is shown with *p* = 0.003. (**C,D**) compare the LTB and HTB groups’ survival curves when using the 2D tumor assessment methods, maximum cross-sectional tumor area, and maximum enhancing tumor area. (**C**) The LTB group includes patients with a tumor burden of ≤ 50.0 cm^2^ for maximum cross-sectional area. The log-rank test showed no significant separation between survival curves of the LTB and HTB groups (*p* = 0.15). (**D**) For maximum enhancing tumor area, categorization into the LTB group was conducted when the maximum enhancing tumor area was ≤ 25.0 cm^2^. The log-rank test demonstrates a *p* = 0.03 between survival curves for the LTB and HTB groups. (**E**,**F,G**) Present Kaplan–Meier curves when using 3D quantitative tumor analysis. (**E**) Categorizing LTB according to assessment of total tumor volume was done when tumor burden was ≤ 410 cm^3^. Log-rank test showed a *p* = 0.03 between Kaplan–Meier curves of LTB and HTB. (**F**) The LTB group was composed of ICC patients with enhancing tumor volume of ≤ 275 cm^3^ (*p* = 0.01 between survival curves of the LTB and HTB groups). (**G**) When using enhancing tumor burden, patients were stratified to LTB when enhancing tumor burden was ≤ 10.0%. A *p* = 0.11 was achieved in the log-rank test between survival curves of the LTB and HTB groups. MOS: Median overall survival.

When using overall tumor diameter with a cutoff of 9.32 cm, the MOS was 1.54 times longer for the LTB group than the HTB group (17.18 months for LTB; 11.17 months for HTB groups, *p* = 0.02). Multivariate analysis showed a hazard ratio (HR) of 0.51 (95%CI: 0.28–0.92; *p* = 0.03) for overall tumor diameter. The mortality ratio of the LTB group was 49% lower than in the HTB group (Fig. 3A).

Based on the largest enhancing tumor diameter with the cutoff of 8.7 cm, the MOS of the LTB group was 1.79 times higher than the MOS of the HTB group (17.18 vs. 9.62 months for LTB and HTB groups, respectively; *p* = 0.003). Comparison of the survival curves between the two groups via the log-rank test revealed significant separation in the survival curves (*p* = 0.003; Fig. 3B). Multivariate analysis showed a HR of 0.46 (95%CI: 0.27–0.78; *p* = 0.004) for maximum enhancing tumor diameter. The mortality ratio for ICC patients with LTB was 54% lower than patients with HTB (Fig. 3B).

#### 2D tumor assessment

The MOS of the LTB group was not significantly different from the HTB group based on the maximum cross-sectional tumor area and cutoff of 51.7 cm^2^ (14.46 vs. 10.97 months for LTB and HTB groups, respectively; *p* = 0.15 on log-rank test), with the multivariate analysis demonstrating a HR of 0.67 (95%CI: 0.40–1.15, *p* = 0.15). Although the LTB group’s mortality ratio was 33% lower than the HTB group (Fig. 3C), it was not statistically significant.

Based on the maximum enhancing tumor area and the cutoff of 25.4 cm^2^, the MOS in the LTB group was 1.79 times higher than the HTB group (17.12 vs. 9.56 months for LTB and HTB, respectively; *p* = 0.03), with a HR of 0.56 (95%CI: 0.33–0.96: *p* = 0.04). Thus, the mortality ratio was 44% lower for the LTB group when compared to the HTB group (Fig. 3D).

#### 3D tumor assessment

The MOS of the LTB group based on the total tumor volume (TTV) cutoff value of 417 cm^3^ was significantly higher than in the HTB group (15.41 vs. 8.15 months, respectively; *p* = 0.03 on log-rank test). This means that the MOS for ICC patients in the LTB group was 1.89 times higher than the HTB group. Multivariate analysis showed a HR of 0.58 (95%CI: 0.34–0.98, *p* = 0.04) for TTV. The mortality ratio in the LTB group was 42% lower than the HTB group (Fig. 3F).

Categorizing ICC patients into the LTB and HTB groups based on the enhancing tumor volume (ETV) cutoff value of 250 cm^3^ via 3D quantitative analysis revealed a MOS of 14.46 months for the LTB group and 9.56 months for the HTB group, respectively. The MOS of the LTB group was 1.51 times higher than the HTB group (*p* = 0.01). There was a significant separation between survival curves of the two groups (*p* = 0.01 on log-rank test). Multivariate analysis showed a HR of 0.52 (95%CI: 0.30–0.91, *p* = 0.02) for ETV, corresponding to a 48% lower mortality ratio for the LTB group when compared to the HTB group (Fig. 3E).

Categorizing the patients into LTB and HTB based on the enhancing tumor burden (ETB) cutoff value of 10.4% demonstrated a MOS of 14.42 months for the LTB group and 9.63 months for the HTB group (*p* = 0.11 on log-rank test). The MOS was 1.50 times longer for the LTB group than the HTB group, though this was not statistically significant. Multivariate analysis showed a HR of 0.65 (95%CI: 0.38–1.10, *p* = 0.11) for ETB. The mortality ratio was 35% lower for the LBT group than the HBT group (Fig. 3G).

### Overview of predictive accuracy

Equality of ROC across methods was evaluated using the DeLong method^25^. While several of the 1D and 2D methods provided a higher area under curve (AUC) corresponding to their ROC curves (i.e., overall tumor diameter, largest enhancing tumor diameter, and maximum enhancing tumor diameter), none of these modalities were statistically greater than the AUC of TTV at the 0.05 level of significance. Moreover, the only discernable difference between the 1D and 2D methods compared to the ETV was the largest enhancing tumor area (*p* = 0.03), while all but the largest tumor area (non-enhancing) were found to have a statistically significant improvement on AUC of ETB. Furthermore, among the 3D modalities, TTV was found to have a statistically great AUC as compared to ETV (*p* = 0.01) and ETB (*p* = 0.01). Lastly, no statistically significant difference was found between the AUCs of ETV and ETB. Thus, of the 3D modalities, TTV and ETV were found to outperform ETB and were comparable to the 1D and 2D modalities.

## Discussion

Our findings showed that the 3D quantitative biomarkers TTV and ETV, as well as the largest overall tumor diameter, enhancing tumor diameter, and maximum enhancing tumor area, on the baseline images are strong predictors of OS in ICC patients who underwent cTACE. Therefore, stratifying ICC patients into the LTB and HTB groups based on TTV and ETV could be utilized to predict which ICC patients would most likely benefit from improved survival following cTACE and guide a more personalized treatment plan for ICC patients.

Our findings also indicated that the largest enhancing tumor diameter, maximum enhancing tumor area, and 3D tumor burden assessment methods focused on TTV and ETV for evaluation of response more reliably predict post-therapeutic outcomes and achieve significant separation of survival curves between patients with LTB and HTB when compared to other tumor assessment methods that do not consider enhancement. There was a significant separation of survival curves when the ICC patients were categorized into the LTB and HTB groups based on the largest enhancing tumor diameter from the modified Response Evaluation Criteria in Solid Tumors (mRECIST, 1D assessment method) and enhancing tumor area calculated based on European Association for the Study of the Liver (EASL, 2D assessment method). These findings are important for practical implementation of tumor assessment, as 1D and 2D techniques are ubiquitous. Similarly, the separation of survival curves was significant when using the 3D quantitative TTV and ETV for categorizing ICC patients into the LTB and HTB groups. The lower HR and good predictive performance of the methods measuring enhancing components of the tumors could be due the fact that the enhancement indirectly reflects the perfusion and vascularity of the tumor. It is well-known that the success rate of the cTACE heavily relies on presence of vascularity for trans-arterial delivery of chemotherapies.

Furthermore, smaller lesions are more likely to be embolized entirely and allow for more complete delivery of chemotherapeutic agents through a limited number of tumor-feeding arteries, compared to larger and more necrotic lesions, which may have multiple tumor-feeding arteries or substantially lower hepatic functional reserve due to larger overall tumor burden. Conceivably, cTACE could result in a more complete necrosis in ICC patients with LTB, which explains the corresponding improved OS in this group. ETV, based on 3D quantitative analysis, is an accurate method of predicting which lesion is most likely to respond to cTACE, as it provides more precise indirect information on the perfusion of the tumor by calculating its enhancement pattern^26^. ETB was found to provide a statistically lower AUC than most of the other modalities; however, TTV and ETV compare very well to the 1D and 2D methods, which, when coupled with the discussed implications on survival in patients receiving cTACE and holistic evaluation of necrosis perfusion, qualifies the 3D modalities as an important clinical tool going forward. Indeed, if confirmed in larger cohorts, 3D quantitative assessment of ICC tumor burden could be used as an early stratification instrument for allocation of ICC patients into LTB and HTB groups, with the former group possibly benefiting the most from cTACE monotherapy. In contrast, the latter group may benefit from combination therapies or systemic chemotherapy.

Although the predictive values of enhancement-based 1D and 2D tumor assessment methods were statistically significant, their inherent imprecision has been reported in prior studies, as these measurement methods lack objectivity and do not reflect the entire tumor volume^17–20^. In order to address these shortcomings, 3D quantitative tumor analysis methods were developed. Three-dimensional quantitative tumor analysis is more reliable and is associated with better inter-reader reproducibility and accuracy in tumors such as hepatocellular carcinoma, metastatic neuroendocrine tumors, metastatic colorectal cancer, metastatic renal cell carcinoma, and now ICC^20,27–30^. Furthermore, 3D quantitative tumor analysis precision for assessment of tumor necrosis in hepatic malignancies treated with TACE has been described in earlier reports^24^. The semi-automated nature of 3D quantitative tumor analysis segmentation software allows it to automatically generate tumor-masks that can then be modified by radiological readers, combining computer-assisted efficacy and human radiological experience^24^. Additionally, the automated subtraction of non-contrast phases from contrast-enhanced MRI phases allows for a more precise and effective assessment of tumor enhancement by mitigating the impact of non-contrast/background hyperenhancement^31^. As volumetric methods rely on enhancement-based functions, 3D tumor quantification can be translated into other diagnostic modalities, including multi-detector CT and intra-procedural cone-beam CT^20^. In this study, a multi-lesion 3D tumor assessment of the entire tumor burden was conducted for every patient to predict outcomes more precisely. Therefore, implementation of computer-assisted 3D quantitative assessment could introduce a new level of workflow-efficiency and clinical relevance for tumor enhancement on baseline imaging when assessing ICC tumor burden.

This study has several limitations. Due to its retrospective nature, the patient populations were inhomogeneous in their disease history, background characteristics, and MRI protocol; in-depth multivariate analysis was performed to reduce the effect of these heterogeneities and counter these limitations. Additionally, this study only evaluated patients treated with cTACE; thus, results and conclusions cannot be applied to ICC patients treated with or considered for other forms of IAT, including bland embolization, drug-eluting beads, transarterial chemoembolization, or radioembolization with Yttrium 90. Future studies could investigate the predictive role of imaging biomarkers for recurrent ICC and lesion-based response.

In conclusion, total tumor volume and enhancing tumor volume show great promise as strong predictors of overall survival in patients with ICC undergoing cTACE. Inclusion of 3D quantitative tumor analysis in guidelines for tumor burden and response assessment in patients with ICC should be considered, given the accuracy and reproducibility of 3D quantitative tumor analysis method.

## Methods and materials

### Study design

This study was conducted retrospectively and received institutional review board (IRB) approval from the Yale School of Medicine. Eligible patients with biopsy-proven ICC lesions and a history of systemic chemotherapy who underwent cTACE were enrolled into this study. Patient records were collected between October 2001 and February 2015. Written informed consent from all subjects was waived by the Yale School of Medicine and Johns Hopkins School of Medicine IRB committees due to the retrospective nature of this study. Data collection and analysis were conducted in compliance with the Health Insurance Portability and Accountability Act (HIPPA). The study design was in agreement with the Standards for Reporting of Diagnostic Accuracy guidelines^32^.

### Study cohort

Clinical and demographic data were obtained from the institutional electronic medical record database. Patients with one or more of the following characteristics were excluded from the study: (1) had unresectable ICC (i.e. patients with mixed ICC and hepatocellular carcinoma were omitted), (2) naïve to locoregional treatment including percutaneous ablations or other intra-arterial therapies than cTACE as well as stereotactic body radiation therapy, and (3) preprocedural multi-phase contrast-enhanced MRI with adequate/artifact-free image quality adequate for 3D quantitative tumor analysis. There were no restrictions on demographic variables, although cofounding variables were adjusted for (see statistical analysis below).

### Clinical and laboratory evaluation and staging

All patients had a complete clinical examination and baseline laboratory workup, including bilirubin, albumin, and International Normalized Ratio (INR). Initial diagnosis of ICC was made on imaging and confirmed on pathology. Furthermore, the Child–Pugh classification for liver function and the ECOG performance status were documented for each patient. The stage of disease was assessed using the Union for International Cancer Control (UICC) staging system.

### cTACE protocol

All patients were discussed in the multidisciplinary tumor-board, and enrolled to undergo cTACE based on final consensus. The interventions were performed by the same interventional radiologist (with more than 20 years of experience) in a dedicated interventional radiology suite (Philips IR suites). After local anesthesia with lidocaine 1%, access was obtained through the common femoral artery via a 5 Fr vascular sheath, followed by a 0.035-in. guide wire using Seldinger technique. For orientation purposes, diagnostic angiography of the superior mesenteric artery and the celiac trunk was obtained using a 5 Fr catheter to selectively advance into the tumor-supplying hepatic artery. Selective catheterization was achieved by placing a microcatheter and obtaining further imaging to target and spare healthy liver parenchyma more precisely. All patients were treated with a cTACE-protocol comprising of a 1:1 mixture of 50 mg doxorubicin (Adriamycin®, Pharmacia & Upjohn) and 10 mg of mitomycin-C with 10 mL of iodized oil (Lipiodol®, Guerbet). This was followed by administration of 100–300 μm diameter microspheres (Embosphere®, Merit Medical); until complete stasis was reached as the technical endpoint.

### Imaging protocol

A standardized liver MRI protocol was performed in all patients enrolled using a 1.5 T scanner (Magnetom Avanto, Siemens) with a phased array torso coil (repetition time ms/echo time ms, 5.77/2.77; a field of view 320–400 mm; matrix 192 × 160; slice thickness 2.5 mm; receiver bandwidth 64 kHz; flip angle 10°). The protocol included single-shot breath-held gradient-echo diffusion-weighted echo-planar images, axial T2-weighted fast spin-echo images, and unenhanced and contrast-enhanced (0.1 mmol/kg intravenous gadopentetate; Magnevist; Bayer) breath-held axial T1-weighted 3D fat-suppressed spoiled gradient-echo images in the hepatic arterial, portal venous and delayed phases (20 s, 70 s, and 180 s, respectively)^24,33,34^.

### Image analysis

All measurements were conducted using standard electronic calipers on Digital Imaging in Communications and Medicine (DICOM) files. Different sequences were assessed to distinguish between tumor enhancement and other hyperintense T1 signal abnormality (such as hemorrhage) in order to evaluate the true extent of tumor burden. If multiple lesions were present, the three largest lesions were assessed, and the sum of total lesions in each patient was then processed in the analysis. For tumor burden analysis using each technique (1D, 2D and 3D), the most dominantly enhancing axial MRI sequence was used in each patient since the enhancement pattern of ICC depends on tumor size and can vary accordingly^35^. The largest overall tumor diameter (1D) and maximum cross-sectional area (2D), as well as the largest enhancing tumor diameter (1D) and maximum enhancing tumor area (2D) were measured by two radiological readers (N.N. with five years of experience and F.L.G with four years of experience in abdominal MRI, respectively), using the RadiAnt DICOM Viewer (Medixant). The numbers used for the final analysis were concluded through consensus between both readers’ simultaneous measurement and discussion; both readers were blinded to all clinical data.

**Figure 4:**
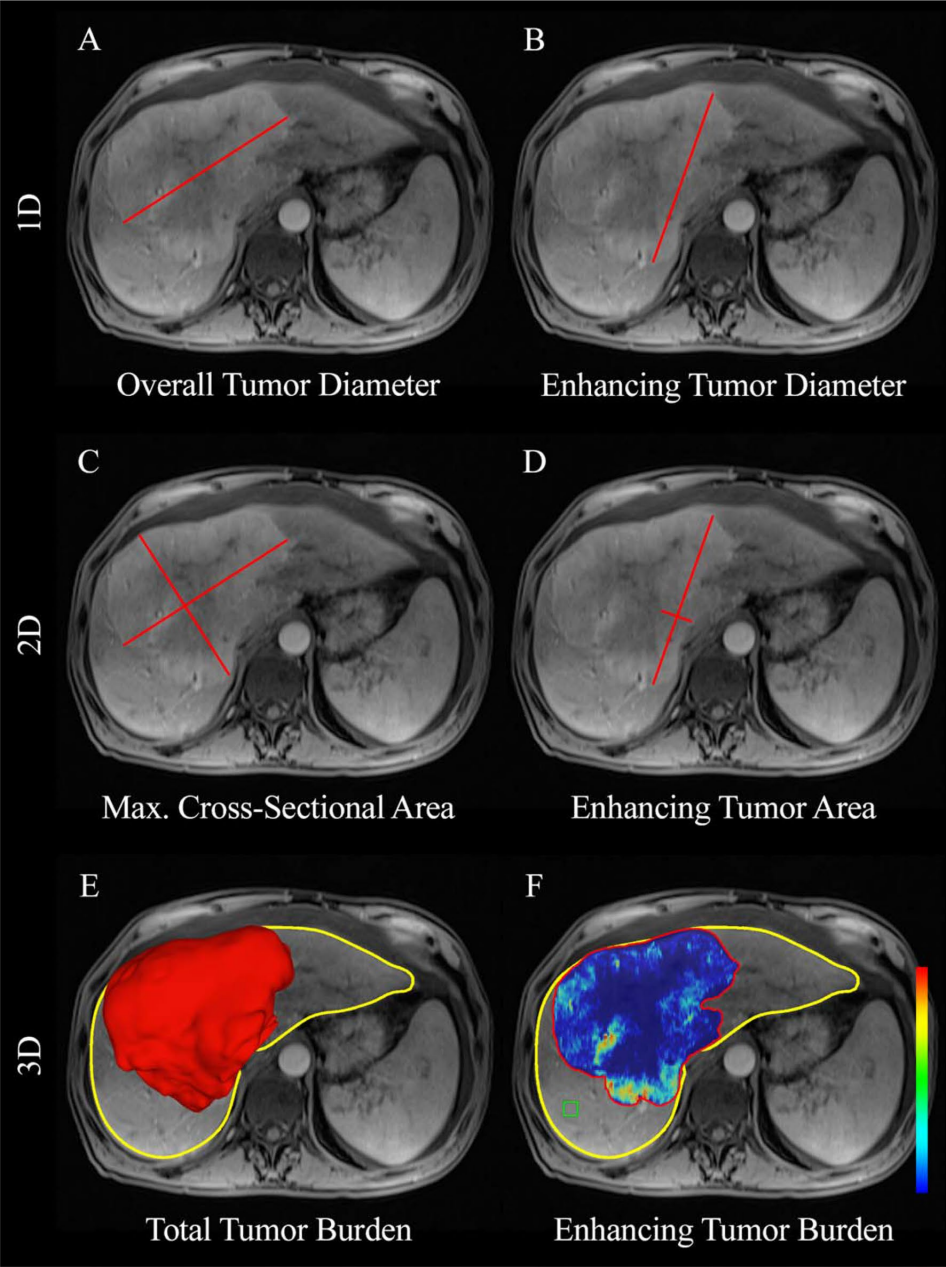
Methods of tumor assessment on enhancement-based MRI. (**A,B**) Demonstrate 1D measurements showing the largest overall tumor diameter (cm) and largest enhancing tumor diameter (cm) represented by the red line. (**C,D**) Show the 2D tumor measurement methods and represent the maximum cross-sectional area (cm^2^) and maximum enhancing tumor area (cm^2^). (**E**) Segmentation of the tumor (3D reconstruction in red) showing the total tumor volume (cm^3^) in relation to the total liver volume (cm^3^), shown by the yellow outline. (**F**) Assessing enhancing tumor volume (cm^3^). Dark-blue regions of the color map represent necrotic tissue; red regions show viable tumor tissue, as described in previous literature^19^. The region of interest (ROI) represented by the green 1 cm^3^ box is used as the relative baseline enhancement of healthy liver parenchyma to calculate the differential enhancement within the segmentation-mask of the tumor.

For the 3D tumor assessment, a 3D quantitative semiautomatic tumor analysis software was used (IntelliSpacePortal V8, Philips ICAP)^19^. The TTV and ETV were assessed by a radiological reader with one year of experience (I.R.). Three-dimensional segmentation-masks of the tumors were created to determine TTV and ETV using quantitative European association for the study of the liver (qEASL) (Fig. 4). The area within the segmentation-mask was considered the TTV and expressed in cubic centimeters (cm^3^) by convention. ETV was assessed using qEASL calculation in cubic centimeters (cm^3^). Initially, axial native MRI T1-weighted images were subtracted from axial enhancing phase images to remove false-positive background enhancement. After subtraction, 3D tumor segmentation-masks were used to select a region of interest (ROI) consisting of a 1 cm^3^ sized cube placed manually within non-tumor liver parenchyma as described previously in the literature^36^. The ROI within the background liver parenchyma sets a cutoff value based on an intensity that is used as a reference to calculate the ETV within the segmentation-masks of the tumors (Fig. 4). After setting the ROI, the software automatically generates a color map of enhancing regions within the segmented 3D tumor mask. The non-enhancing and necrotic areas of the tumor were represented in blue, whereas enhancing and thus viable parts of the tumor were represented in red. The quantitative output resulted in volumetric values indicative of tumor enhancement.

To evaluate the ETB within the liver, the total liver volume (TLV) was calculated using a software prototype (Medisys, Philips Research) that automatically generates a segmentation-mask of the entire liver. The software allows contraction and expansion of the segmentation-mask around control-points within the liver or its contour. Thus, the mask can be manually adjusted by the reader to fully include all anatomical parts of the organ. The true volume of the segmented liver was calculated and enunciated in cubic centimeters (cm^3^). The ETB (%) was calculated using the following formula:

$$\mathrm{ETB }\left(\mathrm{\%}\right)=\frac{\mathrm{ETV}{ (\mathrm{cm}}^{3})}{\mathrm{TLV }{ (\mathrm{cm}}^{3})}\times 100\mathrm{\%}$$

This formula takes into account the ETV in relation to the TLV by calculating their ratio. For comparative purposes, patients were divided into the LTB and HTB groups based on cutoff points defined for each 1D, 2D, ad 3D method.

### Statistical analysis

Statistical analysis of the data was performed using SAS (SAS Institute, Version 9.4.3) and IBM SPSS Statistics (IBM, Version 23.0). Qualitative variables were presented as absolute numbers and percentages. Continuous variables were described by using mean ± standard deviation or median (range). Additionally, the Cox proportional hazard model was used to determine the predictive value of TTV, ETV, and ETB. Survival was calculated based on the interval between the date of embolization and death or the last known alive date. The OS and cumulative survival analysis were calculated and represented using Kaplan–Meier curves, and the log-rank test was utilized to further contrast these survival curves. The Q statistic was used to estimate the most significant cutoff values for each tumor assessment method, then the best area under the curve calculated by ROC curve analysis was confirmed and utilized for every tumor assessment method to determine the optimal cutoff point for patient categorization into LTB and HTB groups, based on improved survival after cTACE. Multivariate analysis adjusted for demographic characteristics, Child–Pugh classification (for liver function), tumor stage, and ECOG (for patient function). Equality of ROC across methods was evaluated using the DeLong method^25^. A *p* value of less than 0.05 was considered statistically significant.

## References

[1] Bergquist A, von Seth E (2015). Epidemiology of cholangiocarcinoma. Best Pract. Res. Clin. Gastroenterol..

[2] Zhang H, Yang T, Wu M, Shen F (2016). Intrahepatic cholangiocarcinoma: Epidemiology, risk factors, diagnosis and surgical management. Cancer Lett..

[3] Wirth TC, Vogel A (2016). Surveillance in cholangiocellular carcinoma. Best Pract. Res. Clin. Gastroenterol..

[4] Rizvi S, Khan SA, Hallemeier CL, Kelley RK, Gores GJ (2018). Cholangiocarcinoma—Evolving concepts and therapeutic strategies. Nat. Rev. Clin. Oncol..

[5] Blechacz B (2017). Cholangiocarcinoma: Current knowledge and new developments. Gut Liver.

[6] Rizvi S, Gores GJ (2013). Pathogenesis, diagnosis, and management of cholangiocarcinoma. Gastroenterology.

[7] Massarweh NN, El-Serag HB (2017). Epidemiology of hepatocellular carcinoma and intrahepatic cholangiocarcinoma. Cancer Control.

[8] Doherty B, Nambudiri VE, Palmer WC (2017). Update on the diagnosis and treatment of cholangiocarcinoma. Curr. Gastroenterol. Rep..

[9] Ierardi AM (2017). The role of interventional radiology in the treatment of intrahepatic cholangiocarcinoma. Med. Oncol..

[10] Razumilava N, Gores GJ (2014). Cholangiocarcinoma. Lancet.

[11] Valle J (2010). Cisplatin plus gemcitabine versus gemcitabine for biliary tract cancer. N. Engl. J. Med..

[12] Vogl TJ (2012). Transarterial chemoembolization in the treatment of patients with unresectable cholangiocarcinoma: Results and prognostic factors governing treatment success. Int. J. Cancer.

[13] Park SY (2011). Transarterial chemoembolization versus supportive therapy in the palliative treatment of unresectable intrahepatic cholangiocarcinoma. Clin. Radiol..

[14] Ma J (2017). Intraarterial liver-directed therapies: The role of interventional oncology. Ochsner. J..

[15] Nezami N (2018). Y-90 radioembolization dosimetry using a simple semi-quantitative method in intrahepatic cholangiocarcinoma: Glass versus resin microspheres. Nucl. Med. Biol..

[16] Nezami N, Camacho JC, Kokabi N, El-Rayes BF, Kim HS (2019). Phase Ib trial of gemcitabine with yttrium-90 in patients with hepatic metastasis of pancreatobiliary origin. J. Gastrointest. Oncol..

[17] Hickey R (2013). Cancer concepts and principles: Primer for the interventional oncologist-part I. J. Vasc. Interv. Radiol..

[18] Stroehl YW, Letzen BS, van Breugel JM, Geschwind JF, Chapiro J (2017). Intra-arterial therapies for liver cancer: Assessing tumor response. Expert Rev. Anticancer Ther..

[19] Lin M (2012). Quantitative and volumetric European Association for the Study of the Liver and Response Evaluation Criteria in Solid Tumors measurements: Feasibility of a semiautomated software method to assess tumor response after transcatheter arterial chemoembolization. J. Vasc. Interv. Radiol..

[20] Tacher V (2013). Semiautomatic volumetric tumor segmentation for hepatocellular carcinoma: Comparison between C-arm cone beam computed tomography and MRI. Acad. Radiol..

[21] Savic LJ, Chapiro J, Geschwind JH (2017). Intra-arterial embolotherapy for intrahepatic cholangiocarcinoma: Update and future prospects. Hepatobiliary Surg. Nutr..

[22] Miyayama S (2019). Ultraselective conventional transarterial chemoembolization: When and how?. Clin. Mol. Hepatol..

[23] Vogl TJ, Gruber-Rouh T (2019). HCC: Transarterial therapies—What the interventional radiologist can offer. Dig. Dis. Sci..

[24] Chapiro J (2014). Radiologic-pathologic analysis of contrast-enhanced and diffusion-weighted MR imaging in patients with HCC after TACE: Diagnostic accuracy of 3D quantitative image analysis. Radiology.

[25] DeLong ER, DeLong DM, Clarke-Pearson DL (1988). Comparing the areas under two or more correlated receiver operating characteristic curves: A nonparametric approach. Biometrics.

[26] Tacher V (2016). Comparison of existing response criteria in patients with hepatocellular carcinoma treated with transarterial chemoembolization using a 3D quantitative approach. Radiology.

[27] Fleckenstein FN (2016). 3D Quantitative tumour burden analysis in patients with hepatocellular carcinoma before TACE: Comparing single-lesion vs. multi-lesion imaging biomarkers as predictors of patient survival. Eur. Radiol..

[28] Sahu S (2017). Imaging biomarkers of tumor response in neuroendocrine liver metastases treated with transarterial chemoembolization: Can enhancing tumor burden of the whole liver help predict patient survival?. Radiology.

[29] Chapiro J (2015). Early survival prediction after intra-arterial therapies: A 3D quantitative MRI assessment of tumour response after TACE or radioembolization of colorectal cancer metastases to the liver. Eur. Radiol..

[30] Fleckenstein FN (2016). Renal cell carcinoma metastatic to the liver: Early response assessment after intraarterial therapy using 3D quantitative tumor enhancement analysis. Transl. Oncol..

[31] Duran R (2014). Uveal melanoma metastatic to the liver: The role of quantitative volumetric contrast-enhanced MR imaging in the assessment of early tumor response after transarterial chemoembolization. Transl. Oncol..

[32] Cohen JF (2016). STARD 2015 guidelines for reporting diagnostic accuracy studies: Explanation and elaboration. BMJ Open.

[33] Pandey A (2018). Baseline volumetric multiparametric MRI: Can it be used to predict survival in patients with unresectable intrahepatic cholangiocarcinoma undergoing transcatheter arterial chemoembolization?. Radiology.

[34] Pandey A (2018). Unresectable intrahepatic cholangiocarcinoma: Multiparametric MR imaging to predict patient survival. Radiology.

[35] Ni T (2018). Different MR features for differentiation of intrahepatic mass-forming cholangiocarcinoma from hepatocellular carcinoma according to tumor size. Br. J. Radiol..

[36] Chockalingam A (2016). Radiologic-pathologic analysis of quantitative 3D tumour enhancement on contrast-enhanced MR imaging: A study of ROI placement. Eur. Radiol..

